# A Group Based Key Sharing and Management Algorithm for Vehicular Ad Hoc Networks

**DOI:** 10.1155/2014/740216

**Published:** 2014-01-22

**Authors:** Zeeshan Shafi Khan, Mohammed Morsi Moharram, Abdullah Alaraj, Farzana Azam

**Affiliations:** ^1^University Institute of Information Technology, PMAS Arid Agriculture University, Rawalpindi 46000, Pakistan; ^2^College of Computer and Information Science, Al Imam Muhammed Ibn Saud Islamic University, Riadyh 11187, Saudi Arabia; ^3^Qassim University, Qassim 51431, Saudi Arabia; ^4^Qassim Colleges, Qassim 51452, Saudi Arabia

## Abstract

Vehicular ad hoc networks (VANETs) are one special type of ad hoc networks that involves vehicles on roads. Typically like ad hoc networks, broadcast approach is used for data dissemination. Blind broadcast to each and every node results in exchange of useless and irrelevant messages and hence creates an overhead. Unicasting is not preferred in ad-hoc networks due to the dynamic topology and the resource requirements as compared to broadcasting. Simple broadcasting techniques create several problems on privacy, disturbance, and resource utilization. In this paper, we propose media mixing algorithm to decide what information should be provided to each user and how to provide such information. Results obtained through simulation show that fewer number of keys are needed to share compared to simple broadcasting. Privacy is also enhanced through this approach.

## 1. Introduction

Vehicular ad hoc networks (VANETs) are the most popular application of wireless communication technologies. Vehicle to vehicle [1] and vehicle to roadside [2] enable the passengers to share safety and comfort information [3]. Traffic management, collision avoidance, and safety warnings are the safety applications [4]. The comfort application [5] gives facility to passenger by sharing information like parking or hotel information and petrol or gas station information. Security is also an essential requirement in VANETs. Jayachandran and Manikandan [6] explain communication and its techniques of broadcast that are mainly used in VANETs.

Mobile ad hoc networks (MANETs) are gaining extraordinary attention by researchers as well as by the industrials. Vehicular Ad-hoc Networks (VANETs) are one special application of MANETs. MANETs are infrastructureless networks that do not require any specialized devices for setup. Nodes act as an end user and a router at the same time. Within the radio range, nodes use the wireless medium to communicate with each other. MANETs are extremely useful in the situations in which it is not feasible to set up a huge infrastructure. Ad hoc networks can be quickly deployed with no administrator involvement. It is very difficult to manage a network of millions of vehicular nodes so these reasons contribute to the ad hoc networks being applied to vehicular environments.

Germany is working on FleetNet project that is purely based on VANETs and it is among the biggest projects of the world. Similarly Japan has started a VANET based project with the name of ITS project. VANETs are gaining popularity along the globe. There are few other names of VANETs given by various well-known organizations. These names are intervehicle communication (IVC), dedicated short range communication (DSRC), or WAVE. In future, it is claimed that journey on highway will become more secure and ratio of accidents will dramatically decrease by using VANETs.

While you are driving, you are constantly changing your location. Change of location changes all the surrounding information. This information can be divided into different categories. The most important category is about driver and vehicle safety information like sharp turns, accidents ahead, road conditions, weather forecast, speed limits, traffic jam, jumps, and railway tracks.

Infotainment is another category in which nearby vehicles can share Internet access, play games, and so forth. Providing parking information is another category of  VANET information. Car maintenance information is another category in which vehicles can provide information to each other about automobile workshops, car mechanics, and so forth.

VANET is mostly configured for sharing safety messages from vehicle to vehicle. There are some scenarios in which group communication is also required by the vehicles. In case of police patrolling, car racing, and tour travelling, the group of vehicles will require sharing information without disclosing it to vehicles outside the group. Group formulation, group management, and key exchange are the issues which required special attention before starting group communication. In this paper, we formulated a media mixing technique, through which vehicles formulate and join the groups, exchange the keys, and manage the group in case of joining and leaving the network. Simulation results showed that the proposed technique performs better as compared to existing techniques.

In the next section, work related to data dissemination and broadcasting is reviewed. Problems are also described at the end of the section. The third section provides details about the media mixing algorithm and its working. The fourth section presents simulation results for performance validation of the proposed media mixing technique. Last section concludes the paper and provides few future directions.

## 2. Related Work 

In simple flooding each message is blindly sent to all the nodes of the network. This creates several problems like redundancy and collision [7]. Probabilistic scheme [8] calculates the probability and sends messages on the basis of that probability. If the probability is increased, it works much like flooding [9, 10]. Counter based technique reduces the redundancy of messages [7]. Distance based scheme first calculates the distance between itself and its neighbor vehicles. Then, it compares the distance with threshold. If the distance is greater than a threshold, it forwards the packet; otherwise, it ignores the message [9, 10]. Location based scheme first calculates the coverage area with help of sender location. The vehicle will ignore the packet if area is smaller than a threshold value; otherwise, the packet will be broadcast [11]. Neighbor knowledge methods [12] maintain a table that contains the information of its neighbor node. A vehicle decision depends upon this information to forward message or not. Broadcast can also be done by using trees. But it is not fit for ad hoc networks, due to the dynamic nature.

Urban multihop broadcast protocol (UMB) is proposed to resolves the reliability, broadcast storm, and hidden node problems, without sharing information among the vehicles. Directional broadcast and intersection broadcast are the two main steps of UMB [13]. Source vehicle selects the furthest vehicle for communication in direction broadcast where as in intersection broadcast installed repeaters at road segments forward the packets to destinations. A mobility-centric data dissemination algorithm for vehicular networks (MDDV) is a mobility centric scheme that merges the idea of opportunistic, trajectory, and geographical forwarding [14]. Trajectory based forwarding [15] is a scheme to forward the packets along a predefined curve in dense vehicular ad hoc network. Geographical forwarding is used for routing decisions. To forward packet to the destination, a vehicle broadcasts the packet to a vehicle that is near to the destination [16]. An opportunistic forwarding [17] has three functions (store, copy, and forward), which give rise to epidemic spreading. MDDV enhance the delivery efficiency and solve the broadcast storm problem. But it still has some shortcoming that it does not differentiate between message types and forward surplus messages without knowing their relevance. Relevance based approach is designed for vehicular ad hoc network as the speed of vehicles is very high and they have limited time to exchange message so they forward only relevant and important messages and discard the low priority messages. Relevance based approach methodology [18] is defined as follows: first we compute the relevance value of message with the help of three resources (vehicle context, message context, and information context) and then we allocate the medium to the messages according to their importance. In this way, low priority traffic cannot get the medium more than high priority traffic. Relevance scheme has certain drawbacks that it does not provide internal resorting of packet queue using 802.11e [19] and it considers the ideal situation that all vehicles are reliable and error free, no concept of malicious node, as it is not possible in real scenarios. Multihop vehicular broadcast (MHVB) [7] uses two algorithms, that is, congestion detection and backfire algorithm. With the help of Congestion Detection, it removes surplus messages and sends packet to destination by using Backfire algorithm.

An adaptive broadcast protocol [20] is proposed to improve the message reliability in VANETs scenario. But it still has to face many challenges like hidden node problem and no priority mechanism in VANETs for providing reliable broadcast. Sequence numbers of packets are useful in order to analyze the network congestion. With the help of sequence number, vehicles dynamically adjust the contention window and improve the performance.

Alam et al., in 2006, proposed the idea of narrowcasting. According to them, instead of sending media burst to all the participants of the conference, it should be delivered according to the preferences of the sender and receivers. Each participant can configure his/her preferences that to whom he/she wants to send her voice and to whom she does not want to [20]. Zeeshan et al., in 2011, presented a media mixing algorithm to push multimedia service in IP multimedia subsystem. Their mechanism works on similar parameters but they purely developed it to push multimedia service [21]. Ahmed et al., in 2009, worked on performance evaluation of different broadcast techniques for VANETs [22].

There hardly exists any single solution that allows the VANETs based vehicles to securely form the group, to establish and share the cryptographic keys, and to manage the group in case of joining of new nodes or excluding of existing nodes. So we need a solution that formulates the group of vehicles of similar interest, treats the incoming and outgoing vehicles efficiently, and securely keeps the privacy.

## 3. Proposed Solution and Methodology: Media Mixing Algorithm 

In this paper, we developed a media mixing algorithm to share the keys among the nodes of the ad hoc network. The objective of this research is to share only the relevant information among the users. Moreover, privacy is also considered as a key parameter in this research work. We design three scenarios to elaborate the media mixing algorithm. Scenario one discusses the normal communication among the existing nodes of the ad hoc network when a network is initiated or created. Scenario two covers the situation when a new node joins the network. The third scenario discusses the case in which an existing node leaves the network.

### 3.1. Scenario 1: Network Initiation

At the time of network creation, each and every node sends its preferences to road side unit (RSU) in form of send/receive list by using PKI. This is based on the assumption that the nodes which want to form the group have knowledge about each other. We assume that every node has information about the existence of all other nodes of the network. The other information that nodes share with the RSU is about how to treat the new nodes. Either a node will allow the new incoming nodes to communicate with it or the node will restrict the new node to communicate with it. The RSU applies media mixing algorithm on this send/receive list and formulates a final list that includes who want to communicate with whom. On the basis of this final list, it creates groups and allocates IDs to each group since each node can belong to many groups at a time. Every node has as many keys as many groups it belongs to. These session keys and group IDs are then forwarded to each node using PKI. Each node has one send session key and as many receiving keys as many nodes are in its send list.

**Figure 1:**
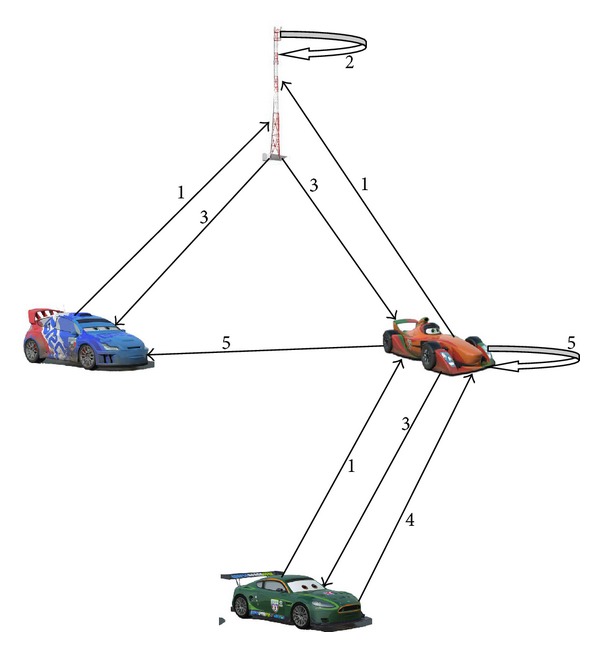
Media mixing algorithm.

Whenever a node wants to send any piece of information, it prepares the message, encrypts it with its send session key, and adds some additional information with it. This additional information includes message ID, group ID, and hash of the first two. At the end, sender signs the message. Accordingly, whenever a node receives a message it first verifies the signature of the sender, and then it checks message ID in order to ensure that previously it has not received the same message. If it finds that it has already received a message with the same ID, the message will be discarded. Otherwise, it will continue processing. Next step is to check the group ID in order to ensure that whether the message belongs to this node or this node is only acting as relay node. For this purpose, every node maintains a history of IDs of received messages up to a specific period of time that may vary from scenario to scenario. If group ID matches with any group ID of the receiver, it means that this message belongs to this node; otherwise, the receiver will simply forward the message. Hash is checked in order to ensure that Message ID and Group ID are not corrupted in transit.  Figure 1 shows the architecture and detailed communication steps involved in media mixing algorithm.

The following are the few abbreviations that we will use in the algorithm below:

 P_b_R: public key of road side unit;
 P_b_N: public key of particular node;
 SSK: send session key;
 P_v_N: private key of a particular node.
Step 1: user sends preferences by encrypting them through public key of RSU (send to/receive from preferences to RSU) P_b_R.
Step 2: RSU applies media mixing algorithm:
  Step 2.1: identify the associated groups of each node;
  Step 2.2: generate session keys;
  Step 2.3: allocate group IDs (group IDs ≤ number of total nodes in the network).
Step 3: send relevant session keys and group IDs to the nodes by encrypting them with each node's public key (session keys + group IDs) P_b_N.
Step 4: a node prepares a message:
  Step 4.1: message is encrypted with send session key (message) SSK;
  Step 4.2: group ID, Message ID, and Hash ID are attached with the message [{(Message)SSK} Group ID, Message ID, and Hash];
  Step 4.3: sender signs the message with its private ID [{(Message)SSK} Group ID, Message ID, and Hash] P_v_N.
Step 5: receiver receives a message:
  Step 5.1: verify signature; if not verified, discard the message; else go to Step 5.2;
  Step 5.2: verify message ID; if it already exists, discard the message; else go to Step 5.3;
  Step 5.3: verify Hash; if not verified, discard the message; else go to Step 5.4;
  Step 5.4: check group ID:
    Step 5.4.1: if ID is matched, decrypt the message and also forward the received message as is;
    Step 5.4.2: if ID is mismatched, forward the packets as it is.

In Table 1, we take a network of 10 nodes. Every node sends its send/receive rules to RSU as shown in the “Send to” and “Receive from” fields of Table 1. The third information is about treating a new incoming node. “N” shows that new nodes are not allowed to communicate while “Y” shows that new nodes are allowed to communicate. The first value in that column shows the “Send to” and the second value shows the “Receive from.”

In Table 2, media mixing algorithm is applied on user's preferences (Send to/Receive from rules of Table 1). In case of clash, priority is given to the Receive from rule whenever a node wants to send data to another node but this node is not willing to receive data from that particular sending node; then the data will not be delivered as the “Receive from” rule has priority over “Send to” rule. After applying the media mixing algorithm we get the results in Table 2. Media mixing algorithm removes all the anomalies existing in Table 1.

In Table 3, we allocate the group IDs to the nodes. The total numbers of groups are equal to the total number of nodes in the network. A single node can be part of multiple groups at a time. In Table 3, we also show how many keys are needed to share with each node of the network. The last column of Table 3 represents this information. It can be seen that user A needs total 8 keys. First key is the “send session key” of the user A which will be used to encrypt any data sent by user A. Since user A is receiving data from 7 other nodes so it needs to share session keys of those 7 nodes with user A. User A will not get session keys of the nodes with whom it does not want to share data like nodes F and P. So any information sent by user F or P will not be delivered to user A because it is not relevant to user A. In this way, we achieved sharing of only the relevant information. On one side, this mechanism saves the valuable resources and on the other side it enhances the privacy within the ad hoc network.

### 3.2. Scenario 2: Joining of a New Node

Whenever a new node joins the networks it also sends its own send/receive preferences to RSU.  Table 4 shows the Send to/Receive from preferences of a new node Z along with the preferences of dealing with new nodes.

After getting the preferences of node Z, the RSU again applies the media mixing algorithm. It scans the “criteria for new node” of all the existing nodes. If a node allows the new node to receive any data from it, then it is matched with the “Receive from” column of the new node in order to ensure whether new node is willing to receive from this node or not. For example, if we look at Table 1, we will find that node A is neither willing to send data to any new node nor willing to receive data from any new node. So node Z cannot have A in its “Send to” and “Receive from” list. Node B is not willing to send any data to a new node but it is ready to receive any data from a new node as shown in Table 1. Media mixing algorithm will check “Send to” preferences of node Z and will find that node Z is willing to send data to node B so node B will be fixed in “Send to” list of node Z. The number of groups and number of keys shared with every node will also change by adding a new node in the network. Table 2 is modified by adding the new node and the final results are shown in Table 5.

### 3.3. Scenario 3: Leaving of an Existing Node

When a node leaves the network, all the keys which are shared with that particular node are refreshed. Moreover, the media mixing algorithm removes the entry of the leaving node from “Send to/Receive from” list of all the other nodes. In Table 6, node D leaves the network, all the keys shared with node D are refreshed immediately and the entry of D is removed from the list of all the existing nodes. Total numbers of keys shared with some nodes are also decreased.

## 4. Results and Discussion

To validate the results and performance of the proposed media mixing algorithm, we simulate it using the NS3. In the first set of experiments, we compared our solution with existing broadcast schemes on the basis of reliability, performance, privacy, relevancy, and redundancy. Results of other techniques are obtained from the literature [23]. Results are summarized in Table 7.

In the best technique, reliability, privacy, and relevancy should be high and redundancy should be very low. The mobility model we use is Manhattan mobility model [24] and generic mobility simulation framework generates the traffic. We find that our solution provides better results in each of the above-mentioned performance criteria as compared to existing broadcast techniques. Simple flooding is highly reliable because a message can reach destination through many different routes. Performance is moderate and congestion is very high in simple flooding due to transmission of every packet to all the neighbors. Since message is delivered through blind broadcasting, privacy cannot be achieved and relevancy cannot be considered in case of simple flooding. Message can reach destination through different routes so redundancy is also very high in simple flooding.

Probabilistic scheme reduces the collision, contention, and redundant messages in dense network as it broadcasts the messages with some fixed probability. But, in sparse network, all the vehicles cannot receive the same packets with small probability. If the probability is increased, it works much like flooding. Hence, its performance becomes greater in dense network as compared to sparse network. Counter base scheme reduces the redundancy while giving the performance similar to probabilistic scheme.

Distance based and location based approaches are the moderate approaches while the neighbor knowledge methods compromise on privacy and are moderate in redundancy. Tree based approach again compromises on privacy while relevancy is also moderate in this approach. UMB and MDDV are average not suitable approaches in terms of privacy and relevancy. Relevance based approach is among the better approaches but privacy and reliability are moderate in this approach. MHVB compromises over privacy while adaptive broadcast protocol is redundant and not suitable in terms of privacy. Our proposed approach is highly reliable because of the extra information attached with the packet. Performance is also very high as key refreshment load is reduced and message is also delivered only to relevant nodes. Privacy is achieved by sharing the key only between those nodes which really want to communicate with each other. Since each user specifies that from whom he/she wants to receive and to whom he/she wants to send so the solution provides high level of relevancy. Message ID and group ID jointly control the redundancy of messages in our proposed solution so redundancy is very low in our solution.

**Figure 2:**
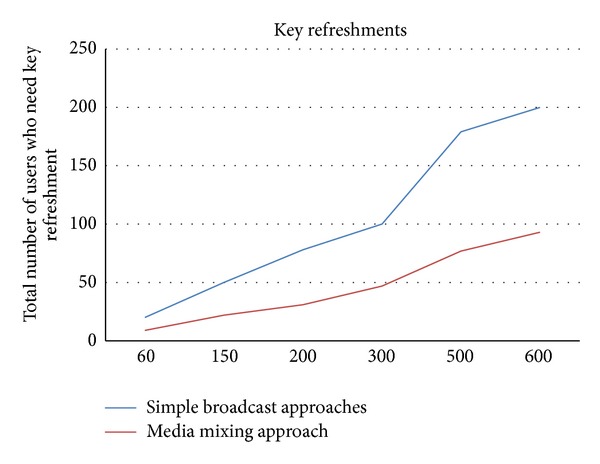
Total number of nodes that need key refreshment.

In the next set of experiments we take total 10 nodes and we measured how many nodes need a refreshed key after certain period of time. We set an environment in which a node leaves the network after every 60 seconds and within these 60 seconds one new node also joins the network. We find that after 600 seconds total 200 nodes refreshed their keys, those have already joined the network without leaving it, in simple broadcast scheme. We repeat the same experiment by implementing our media mixing technique and we find that total 93 nodes refreshed their keys within 600 seconds. Results are shown in Figure 2.

**Figure 3:**
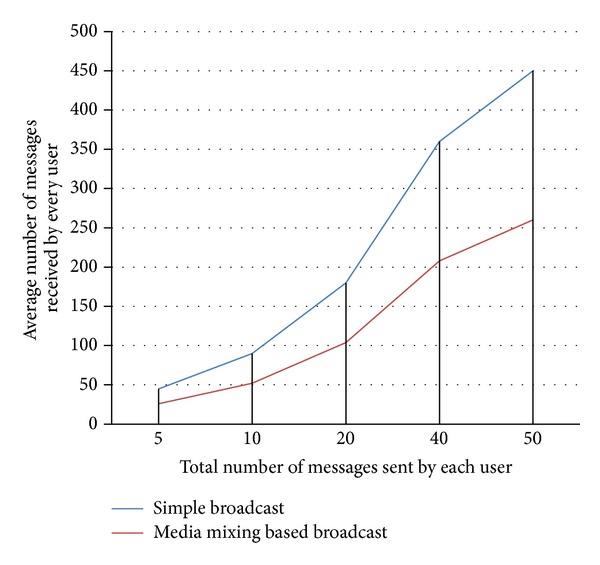
Average number of messages received by each user.

In the second set of experiments, we measured the average number of messages received by every node within the network. As per Figure 3, we used 10 nodes to test the scenario and the results were measured by sending different number of messages. When every node of the network sends 50 messages, the average number of messages received by a single node is 450 in simple broadcast technique while it is 260 in media mixing based broadcast. In simple broadcast scheme 190 extra messages are delivered to every node within the network that consume considerable wastage of resources at receiver's end because these messages are not relevant to that particular users and categorized as unwanted messages. So on one hand these messages create disturbance for the receiver and on the other hand these consume resources.

**Figure 4:**
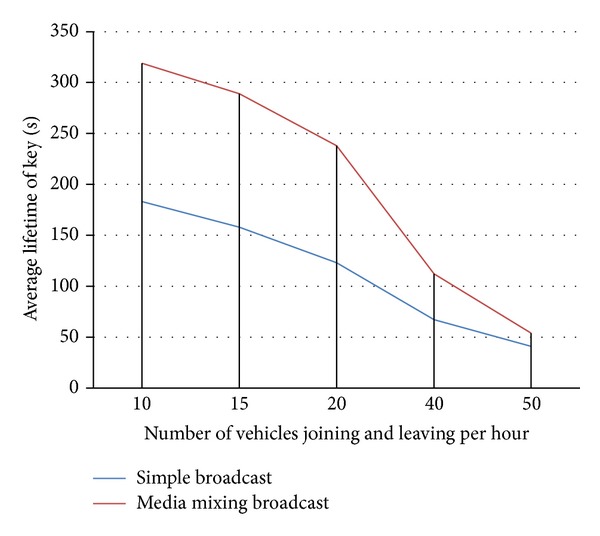
Average lifetime of a key.

In last set of experiments, we measured that how frequently a node needs a refreshed key. 600 seconds are set as maximum life time of a key. After that, we tested the average time by changing the joining and leaving rate of vehicles. When 10 nodes leave and the other 10 nodes join the network within an hour, we find that the key needs to be refreshed after every 183 seconds in simple broadcast while after every 319 seconds in media mixing based broadcast. When 50 nodes per hour are joining and leaving the network, the key needs to be refreshed after every 41 seconds in simple broadcast while after every 54 seconds in media mixing based broadcast technique. So we can conclude that in media mixing based broadcast keys are refreshed less frequently (with respect to maximum life time of the key) as compared to simple broadcast techniques.  Figure 4 shows the above mentioned results.

## 5. Conclusion 

In VANETs, nodes are leaving and joining the network very frequently so it is not preferred to use unicast based protocols. Due to its dynamic topology, broadcast is always preferred in VANETs. Simple broadcasting creates different types of problems including privacy, disturbance, and resource utilization problems. In this paper, we proposed a media mixing algorithm for VANETs that solves these problems and utilizes the cryptographic keys efficiently. Rules are specified by every node and after that a media mixing algorithm is applied on the specified rules and a final list of rules is determined. Keys are distributed on the basis of groups formulated through media mixing algorithm. We have conducted all the experiments by taking lesser number of nodes but by increasing the number of nodes results may change a bit. In future it is planned to test the solution for more dense and populated network scenarios.

## Figures and Tables

**Table 1:** User's preferences.

User	Send to	Receive from	Criteria for new node
A	C, D, F, P, S	B, C, D, E, F, G, Q, P, S	N, N
B	A, C, P, S	A, C, P, S	N, Y
C	A, B, D, F, P, S	A, B, D, E, F, G, Q, P, S	N, Y
D	A, B, C, E, F, G, Q, P, S	A, B, C, F, S	Y, Y
E	A, B, G, P, S	A, B, G, P, S	Y, N
F	B, C, D, G, Q	A, B, D, E, G, Q	Y, Y
G	A, B, C, D, E, F, Q, P, S	A, B, C, D, E, F, Q, P, S	Y, N
Q	A, B, C, D, E, F, G, P, S	A, B, C, D	N, Y
P	B, C, D, F, G	B, C, D, F, G, Q	N, Y
S	A, B, C, D, E, F, G, Q, P	C, F, G, Q	Y, N

**Table 2:** Application of media mixing algorithm on user's preferences.

User	Send to	Receive from
A	C, D, F	B, C, D, E, G, Q, S
B	A, C, P	C, P, S
C	A, B, D, P, S	A, B, D, F, G, Q, P, S
D	A, C, F, G, Q, P	A, C, F, S
E	A, G	G
F	C, D, G	A, D, G, Q
G	A, C, E, F, P, S	E, F, Q, P, S
Q	A, C, F, G, P, S	D
P	B, C, G	B, C, D, G, Q
S	A, B, C, D, E, G	C, G, Q

**Table 3:** Group IDs and session keys for each node.

User	Group ID	Send to	Receive from	Session keys
A	1	C, D, F	B, C, D, E, G, Q, S	1 + 7
B	2	A, C, P	C, P, S	1 + 3
C	3	A, B, D, P, S	A, B, D, F, G, Q, P, S	1 + 8
D	4	A, C, F, G, Q, P	A, C, F, S	1 + 4
E	5	A, G	G	1 + 1
F	6	C, D, G	A, D, G, Q	1 + 4
G	7	A, C, E, F, P, S	E, F, Q, P, S	1 + 5
Q	8	A, C, F, G, P, S	D	1 + 1
P	9	B, C, G	B, C, D, G, Q	1 + 5
S	10	A, B, C, D, E, G	C, G, Q	1 + 3

**Table 4:** Send to/receive from preferences of a new node Z.

User	Send to	Receive from	Criteria for new node
Z	A, B, C, D, E, P, S	A, B, C, D, P, S	Y, Y

**Table 5:** Group IDs and session keys for each node after joining of a new node.

User	Group ID	Send to	Receive from	Session keys
A	1	C, D, F	B, C, D, E, G, Q, S	1 + 7
B	2	A, C, P	C, P, S, Z	1 + 4
C	3	A, B, D, P, S	A, B, D, F, G, Q, P, S, Z	1 + 9
D	4	A, C, F, G, Q, P	A, C, F, S, Z	1 + 5
E	5	A, G	G	1 + 1
F	6	C, D, G	A, D, G, Q	1 + 4
G	7	A, C, E, F, P, S	E, F, Q, P, S	1 + 5
Q	8	A, C, F, G, P, S	D	1 + 1
P	9	B, C, G	B, C, D, G, Q, Z	1 + 6
S	10	A, B, C, D, E, G	C, G, Q	1 + 3
Z	11	B, C, D, P	D, S	1 + 2

**Table 6:** Send to/receive from list and keys information after a node leaves the network.

User	Send to	Receive from	Session keys
A	C, F, P, S	B, C, E, G, Q, S	1 + 6
B	A, C, P, S	C, P, S, Z	1 + 4
C	A, B, F, P, S	A, B, F, G, Q, P, S, Z	1 + 8
**D**	**A, B, C, E, F, G, Q, P, S**	**A, C, F, S, Z**	**1 + 5**
E	A, B, G, P, S	G	1 + 1
F	B, C, G, Q	A, G, Q	1 + 3
G	A, B, C, E, F, Q, P, S	E, F, Q, P, S	1 + 5
Q	A, B, C, E, F, G, P, S		1
P	B, C, F, G	B, C, G, Q, Z	1 + 5
S	A, B, C, E, F, G, Q, P	C, G, Q	1 + 3
Z	B, C, P	S	1 + 1

**Table 7:** Comparison of media mixing algorithms with existing broadcast approaches.

Protocols	Reliability	Performance	Privacy	Relevancy	Redundancy
Simple flooding	Very high	Moderate	Very low	Very low	Very high
Probabilistic scheme	Moderate	Moderate	Low	Moderate	Moderate
Counter based scheme	Moderate	Moderate	Very low	Low	Low
Distance based approach	Moderate	Moderate	Moderate	Moderate	Moderate
Location based approach	Moderate	Moderate	Very low	Moderate	Moderate
Neighbor knowledge method	High	High	Very low	High	Moderate
Tree based broadcast	High	High	Very low	Moderate	Low
UMB	High	High	Very low	Low	Moderate
MDDV	High	High	Very low	Very low	Low
Relevance based approach	Moderate	High	Moderate	High	Low
MHVB	High	High	Very low	Moderate	Low
Adaptive broadcast protocol	High	High	Very low	Moderate	High
Media mixing algorithm (proposed)	High	High	High	High	Very low
